# The Effects of Clinical Hypnosis versus Neurolinguistic Programming (NLP) before External Cephalic Version (ECV): A Prospective Off-Centre Randomised, Double-Blind, Controlled Trial

**DOI:** 10.1155/2012/626740

**Published:** 2012-06-21

**Authors:** Joscha Reinhard, Swati Peiffer, Nicole Sänger, Eva Herrmann, Juping Yuan, Frank Louwen

**Affiliations:** ^1^Department of Obstetrics and Gynaecology, Faculty of Medicine, Johann Wolfgang Goethe University of Frankfurt am Main, Theodor-Stern-Kai 7, 60590 Frankfurt am Main, Germany; ^2^Institute of Biostatistics and Mathematical Modeling, Faculty of Medicine, Johann Wolfgang Goethe University of Frankfurt am Main, Theodor-Stern-Kai 7, 60590 Frankfurt am Main, Germany

## Abstract

*Objective*. To examine the effects of clinical hypnosis versus NLP intervention on the success rate of ECV procedures in comparison to a control group. *Methods*. A prospective off-centre randomised trial of a clinical hypnosis intervention against NLP of women with a singleton breech fetus at or after 37^0/7^ (259 days) weeks of gestation and normal amniotic fluid index. All 80 participants heard a 20-minute recorded intervention via head phones. Main outcome assessed was success rate of ECV. The intervention groups were compared with a control group with standard medical care alone (*n* = 122). *Results*. A total of 42 women, who received a hypnosis intervention prior to ECV, had a 40.5% (*n* = 17), successful ECV, whereas 38 women, who received NLP, had a 44.7% (*n* = 17) successful ECV (*P* > 0.05). The control group had similar patient characteristics compared to the intervention groups (*P* > 0.05). In the control group (*n* = 122) 27.3% (*n* = 33) had a statistically significant lower successful ECV procedure than NLP (*P* = 0.05) and hypnosis and NLP (*P* = 0.03). *Conclusions*. These findings suggest that prior clinical hypnosis and NLP have similar success rates of ECV procedures and are both superior to standard medical care alone.

## 1. Introduction

At full-term singleton 3-4% of pregnancies present themselves as breech deliveries [1, 2]. External cephalic version (ECV) is a procedure to try to turn a breech fetus to cephalic by externally manoeuvring the fetus through the maternal abdomen. ECV decreases the likelihood that the fetus will be in a noncephalic presentation at birth and the need for caesarean section [3–6]. Without contraindication, ECV should be recommended for all women with a breech fetus at term [3–6]; however, ECV is only successful in about 40% of attempts [4, 7–9].

During labour and other medical procedures clinical hypnosis is an effective method to reduce pain und distress [10–17]. Pregnant women demonstrated highest suggestibility for trance when compared with pregnancy and postpartum period [18]. A clinical hypnosis intervention can also reduce the muscle tone [15, 19, 20]. Our study group could recently show that a prior clinical hypnosis intervention can increase the success rate of ECV when compared to standard medical care [21].

The goal of this study is to compare the ECV success rate of a clinical hypnosis intervention versus a NLP intervention. A secondary goal is to compare the ECV success rate of the intervention with a control group of standard medical care alone.

## 2. Methods

### 2.1. Study Design

This was a single centre, stratified (parity), double-blind, parallel-group (clinical hypnosis or NLP) study conducted in a tertiary university hospital in Germany.

Enrollment of the participants was done by J. R. A off-centre randomisation sequence based on a block randomisation was calculated and assigned by the Institute of Biostatistics and Mathematical Modeling (E. H.). The study received ethical approval at the local ethics committee and has been registered at clinical trials (NCT01564004).

Eligible participants were pregnant women with a singleton fetus in a breech position at the scheduled date of the ECV at or after 37^0/7^ (259 days) weeks of gestation, normal amniotic fluid index, and with advanced level of German language. The only exclusion criteria were in active labour patients (regular uterine contractions, and rupture of membranes), contraindications for a vaginal birth (such as placenta praevia), and a planned birth by caesarean section even if the fetus turned to a cephalic position. The ECV procedures were undertaken by J. R. an experienced clinician in ECV, who is head of division, agreed with that judgment [22].

The study took place at a tertiary referral centre of the Johann Wolfgang Goethe University Hospital in Frankfurt am Main, Germany, from January 2009 to December 2011. The standard medical care did not change during the whole study period and no changes to methods after trial commencement.

### 2.2. Control Group

From January 1, 2009 to October 31, 2010, a control group were all ECVs, during which time neither hypnosis nor NLP was used.

### 2.3. The Intervention

In November 1, 2010, the initiation of this study took place. A screening ultrasound was undertaken before the ECV procedure. If the patient consented to take part in the study, women were randomly assigned to hear a hypnosis or NLP intervention (ca. 20-minute standardized clinical hypnosis or NLP intervention via head phones (Bose, QuietComfort 15) before ECV procedure was carried out. The hypnosis intervention was a voice recording of J. R., who is also a certified hypnotherapist and underwent training in the fundamentals of NLP.

For the hypnosis intervention, a relaxation induction was utilized, in which the therapist focused on the breathing as well as concentrating on various parts of the body for trance deepening. The suggestion of the “smiling child” after Lorenz-Wallacher [23] was used: “… While you can allow yourself to enjoy the relaxation and felling of wellbeing … which is spreading more and more … you might would like to imagine … how a muscle, organ or tissue is starting to smile … and since smiling is contagious … some region of you is smiling back … and you might start to feel … how you are sensing when the smiling is spreading … more and more spreading throughout your body … in the whole body … downwards towards the uterus … and the uterus is starting to smile … and each muscle fiber is smiling and relaxing … the uterus is smiling to the baby all around … the uterus smiling towards your baby at the center … from all sides smiling and relaxing … more and more … softer and softer … each muscle fiber relaxing, lengthening … smiling … and you might feel … how your baby is reacting … when your baby is receiving the friendly smiles all around … Can you see how you baby is smiling back? … Maybe you would like to imagine … in your imagination to smile towards your baby … and tell your baby all those kind words, which you would like to tell your baby … let your baby feel all your love … to enjoy feeling save and secure … saver, more and more secure … and if you want to you can get into contact with your baby … With your imaginative hands you can stroke and touch your baby … Can you feel the baby … You can tell your baby how you are looking forward to the arrival of your baby at the right time … and maybe your baby would like to tell you something … … and you can enjoy the contact with your baby … the connection between you and your baby … and you can contact your baby now … and in the future whenever you want to … smiling … relaxing … more and more … I wonder where in your body you can already feel this relaxation … This relaxation may grow with every breath … Your baby can flow freely … turn freely …. “Dehypnotization proceeded by backward counting. During this process, suggestions were given to the effect that the patient would be relaxed and smiling even when not thinking about it [21].

The intervention was double-blinded that is the participant and the clinician, who is carrying out the ECV procedure, did not know the kind of intervention.

30 minutes prior to the ECV procedure, fetal wellbeing was assessed by continuous fetal heart rate monitoring, and a low concentration of tocolytic agents to relax the uterus was started. A maximum of three ECV procedures were carried out. The ECV was discontinued if it was not easily accomplished, or if the woman reported undue discomfort or the fetal heart rate was nonreassuring. Fetal presentation was confirmed by ultrasound directly after ECV procedure and before discharge by an independent senior house officer. All women were monitored for at least 60 minutes as well as 3 hours after ECV procedure for another 60 minutes. Anti-D immunoglobulin was recommended for all rhesus-negative women following the procedure.

### 2.4. Outcome Measures

The primary endpoint with respect to efficacy in ECV was the proportion of patients with cephalic presentation 4–6 hours after ECV procedure verified using ultrasound examination (successful ECV). Additional analyses were done on standardized questionnaires of 53 items 30–60 minutes after the ECV procedure. Six answer options were available: 1: complete agreement, 2: agreement, 3: slight agreement, 4: slight disagreement, 5: disagreement, and 6: complete disagreement.

### 2.5. Sample Size

The power calculation is based on the assumption that 60% of the population are primiparas, and they have a 50% ECV success rate. Multiparas are assumed to have a 60% ECV success rate. An odds ratio of success rate of clinical hypnosis and NLP is assumed to be 1.6. Hence, 716 women need to be included for a statistical power of at least 80%.

### 2.6. Statistical Analysis

For *statistical analyses,* the Welch, Mann-Whitney, and Fisher exact tests were applied. The analyses were carried out using the SPSS Statistics 17.0 software. The means and standard deviation (SD) were processed. *P* < 0.05 for a two-tailed test was considered statistically significant.

## 3. Results

**Figure 1:**
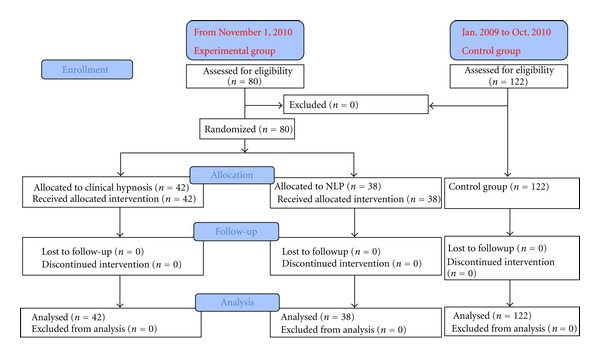
Flow Diagram.

All 80 patients gave written informed consent to the study (Figure 1). During the study period, no patient agreed to enter the study. Baseline characteristics (size, weight before pregnancy, current weight, gestation age, amniotic fluid index, fetal weight estimation, parity, and breech firmly fixed in the pelvis) were similar in the two groups (*P* > 0.05; Tables 1 and 2). The success of the ECV procedure and complication rates are presented in Table 3. Of the 42 women in the hypnosis group, 40.5% (*n* = 17) had a successful ECV procedure, whereas 44.7% (*n* = 17) of the NLP group (*n* = 38) had a successful ECV procedure (*P* > 0.05).

Of the 122 women in the control group, 27.3% (*n* = 33) had a successful ECV procedure. Statistically improved success rates were seen when comparing NLP (*P* = 0.05) or hypnosis and NLP together (*P* = 0.03) with the control group (Table 3); however, hypnosis only had a trend to higher success rates when compared to the control group (*P* = 0.08).

The standardised questionnaire demonstrated a statistically significant difference (*P* < 0.05) only in the following parameters: NLP patient group felt slightly better supported by the doctor/midwife (*Z* = 2.1; *P* = 0.04) and slightly more relaxed during ECV (*Z* = 2.1; *P* = 0.04) (Table 4). All other items (pain, discomfort, etc.) did not demonstrate a statistically significant difference.

All important harms or unintended effects in each group have not been observed.

## 4. Comment

At term ECV reduces the need for caesarean section and is considered safe for the fetus [3, 7, 8, 24, 25]. Using clinical hypnosis compared with standard medical care the likelihood of successful ECV increases [21], thereby further reducing the need for caesarean section.

This trial found no difference of ECV success rate between a hypnosis intervention and NLP; however, if comparing with standard medical care a statistically significant improvement to standard medical care was demonstrated for NLP. Hypnosis did not reach statistically significant difference. This can be explained with the low patient numbers. A previous lager hypnosis trial has already demonstrated improved ECV success rate of hypnosis when compared to standard medical care [21]. Similar scores were found for NLP and hypnosis intervention of the questionnaire items (55 items) for pain, discomfort, feeling save, anxiety, recommendation of ECV to a best friend, and relaxation due to the intervention.

Generally most women are stressed in a hospital environment and go into a “natural” hypnotic state, hence calming and relaxing the women is generally helpful [26] and has also been shown in this study.

The strength of this study is the prospective off-centre randomised, double-blind trial design; however, the weakness of this study is the low patient numbers and the potential biased of a nonrandomized control group. The number of normal vertex birth has not been analysed since most patients delivered in their local hospital and not in our tertiary centre.

The use of complementary medicine and alternative medicine is frequently applied during pregnancy [27]. A positive effect of complementary and alternative medicine has been described [10, 28]; however, further prospective randomised trials are required.

## 5. Conclusions

In conclusion, for the first time the results of the present study indicate that clinical hypnosis and NLP have no difference in ECV success rates, and hence both can increase the success rates if compared to standard medical care [21] alone.

## Figures and Tables

**Table 1:** Baseline characteristics (mean ± standard deviation) (Welch test, ns = *P* > 0.05).

Characteristic at randomisation	NLP (*n* = 38)	Hypnosis group (*n* = 42)	Control group (*n* = 122)	*P* value
Maternal height (cm)	169 ± 7.4	168.9 ± 6.3	167.2 ± 5.6	ns
Maternal weight before pregnancy (kg)	63.6 ± 12.6	63.9 ± 8.5	68.3 ± 13.9	ns
Current maternal weight (kg)	76.9 ± 13.6	77.9 ± 9.4	—	ns
Gestation age	37.2 ± 0.4	37.2 ± 0.6	37.9 ± 3.3	ns
Amniotic fluid index (cm)	13.6 ± 3.6	13.0 ± 3.6	—	ns
Fetal weight (g)	2850 ± 297	2819 ± 270	—	ns

**Table 2:** Baseline characteristics of parity and firmness of (ns = *P* > 0.05).

Characteristic at randomisation	NLP (*n* = 38) (%)	Hypnosis group (*n* = 42) (%)	Control group (*n* = 122) (%)	*P* value
Parity				
(i) 0	25 (65.8)	30 (71.4)	81 (66.4)	ns
(ii) ≥1	13 (34.2)	12 (28.6)	41 (33.6)	ns

Breech firm in the pelvis				
(i) Firm	18 (47.4)	28 (66.7)	—	ns
(ii) Not firm	20 (52.6)	14 (33.3)	—	ns

**Table 3:** Percentage of successful ECV procedures (numbers).

		Control group (*n* = 122)	*P* value
NLP (*n* = 38)	44.7% (17)	27.3% (33)	0.05
Hypnosis (*n* = 42)	40.5% (17)	27.3% (33)	0.08
NLP and Hypnosis (*n* = 80)	42.5% (34)	27.3% (33)	0.03

**Table 4:** Mean ± standard deviation of number of ECVs for each participant and duration time, pain relief during ECV manoeuvre, and a selection of questionnaire (^∗^) items for the hypnosis (*n* = 42) and NLP (*n* = 38) intervention.

	NLP	Hypnosis group *n* (%)	*P* value
Number of ECV for each participant	2.3 ± 1.0	2.2 ± 0.9	ns
Duration of ECV (minutes)	5.9 ± 3.4	6.0 ± 3.4	ns
Good pain relief during ECV	3.3 ± 1.8	3.4 ± 1.7	ns
Wish for more pain killers during ECV	4.8 ± 1.5	5.4 ± 1.2	ns
Strong pain during ECV	3.6 ± 1.9	3.6 ± 1.8	ns
Negative memories	5.4 ± 1.1	5.2 ± 1.3	ns
ECV was as expected	2.7 ± 1.7	2.8 ± 1.8	ns
I felt safe during the ECV	1.8 ± 0.8	1.8 ± 0.8	ns
I had everything under control	2.5 ± 1.2	2.4 ± 1.3	ns
I would recommend a ECV to my best friend	1.4 ± 0.9	1.7 ± 1.2	ns
I have good memories of the ECV	2.8 ± 1.8	3.4 ± 1.7	ns
Intervention (hypnosis or NLP) was helpful	1.6 ± 1.1	2.0 ± 1.7	ns
Intervention (hypnosis or NLP) was relaxing	1.4 ± 0.9	2.2 ± 1.7	0.04
Good support by the doctor/midwife	1.1 ± 0.4	1.3 ± 0.6	0.04

*1: absolutely true, 2: mainly true, 3: slightly true, 4: slightly not true, 5: mainly not true, 6: absolutely not true.
